# A Heatmap-Based Risk–Benefit Assessment of Traditional and Processed Meat Products

**DOI:** 10.3390/foods15040661

**Published:** 2026-02-12

**Authors:** Erfan Bagherzadehsurbagh, Marta Laranjo, Ricardo Assunção

**Affiliations:** 1 Department of Food Engineering, Graduate School of Natural and Applied Sciences, Akdeniz University, Pinarbasi, Konyaalti 07070, Antalya, Türkiye; bagherzadeh.erfan@gmail.com; 2 MED-Mediterranean Institute for Agriculture, Environment and Development & CHANGE-Global Change and Sustainability Institute, Departamento de Medicina Veterinária, Escola de Ciências e Tecnologia, Universidade de Évora, 7004-516 Évora, Portugal; mlaranjo@uevora.pt; 3 Food and Nutrition Department, National Institute of Health Dr. Ricardo Jorge, 1649-016 Lisboa, Portugal; 4 Egas Moniz Center for Interdisciplinary Research (CiiEM), Egas Moniz School of Health & Science, 2829-511 Caparica, Portugal

**Keywords:** risk–benefit assessment, pork meat, fermented meat products, nutritional composition, microbiological hazards, chemical hazards, public health

## Abstract

Meat and meat products are integral components of various diets and provide many macro- and micronutrients. However, concerns over their potential adverse health effects remain pressing. This study employed a semi-quantitative Risk–Benefit Assessment (RBA) methodology to compare both beneficial and adverse health impacts of various meat products, i.e., fermented (Salame and Chouriço), dry-cured (Presunto), and heat-treated (Fiambre), with unprocessed grilled pork meat as a reference. Nutritional composition and microbiological and toxicological hazards were assessed using data acquired from national and international databases and the literature. In the end, a heatmap approach was used to summarize and compare product profiles. While grilled pork offered the most favorable profile, processed products showed high levels of sodium, nitrites, and contaminants, such as polycyclic aromatic hydrocarbons and ochratoxin A. Notably, Salame exhibited the most concerning risk profile, including high levels of histamine and ochratoxin A, whereas Fiambre, despite the high nitrite content, showed the lowest microbial and toxicological risks. These findings highlight significant variability in health-related impacts among meat products, caused mainly by processing technologies. The results can improve dietary guidance and regulations and encourage innovations, especially by indicating the potential of using engineered fermentation techniques and novel additives for improved meat products.

## 1. Introduction

Meat is one of the most used sources of protein, alongside many other micronutrients, such as vitamins, fatty acids and iron in the human diet, and one of the most consumed foods worldwide [1,2]. However, there are many debates in the scientific community regarding the beneficial and adverse health effects of the human consumption of red meat and its products, particularly regarding the dose–response relationships and threshold effects, and the effects of processing/cooking methods on the health effects of red meat and other subjects [3]. Red meat consumption, particularly in the form of processed meat products, has been associated with various health concerns, such as colorectal cancer [4]. Consequently, in recent years, a decrease in meat consumption has been promoted and encouraged by some national and international dietary guidelines [5] and, as stated, there are concerns over the role of meat consumption in many health-related concerns and diseases [6]. On the other hand, an increase in demand for protein as a whole, and specifically meat, has been verified [1]. This increase in demand is a consequence of the rising worldwide population and an increase in income levels of low- or middle-income countries and certain socioeconomic groups. In countries with an immensely growing middle-income class, such as Brazil, China and India, the demand for meat and meat products has increased and is predicted to rise even more, due to elevating levels of income and urbanization, which drives the shift towards more protein-rich foods such as meat, which are traditionally associated with wealth [7]. In Europe, however, especially in western European countries such as Germany, France and the Netherlands, there has been a decrease in the demand for meat and meat products, and more consumers are leaning towards plant-based alternatives, reducing their meat intake or modifying their diet as a whole. While this is the case in these countries, in other parts of Europe, such as Southern European countries like Spain and Portugal, there is much less preference shift from meat products, especially among older adults [8,9]. In general, despite many efforts being devoted to lowering meat and meat product consumption, due to both environmental burden and health concerns, global consumption is increasing. Hence, it is important to understand the complex health-related influences of meat and meat product consumption worldwide and in Europe.

Meat is one of the most perishable food items, so different cultures around the world have developed various practices to preserve this food throughout history. These methods, which utilize mutual basic techniques such as drying, smoking, salting and fermenting, have led to various types and categories of meat products in different parts of the world. For instance, fermented meat products, such as Salami (Italy), Sucuk (Türkiye) and Chouriço (Spain), are all prepared in similar ways with minor differences in their recipe, formulation or preparations. In general, these products are made by mincing certain ratios of fat and lean meat, mixing them with ingredients such as salt and spices (e.g., oregano, cumin, garlic peppers or paprika, which is an important characteristic of Chouriço), to form the batter, and then filling them in casings and leaving them for fermentation and ripening. The differences between these products and between subtypes of these products are mainly the minor details of the production process, which vary substantially depending on region, tradition, and even family shops for artisanal types [10]. Applying the smoking process in these products also depends on the region of production. For instance, Chouriço produced in the northern parts of Spain are usually smoked, which prevents mold and improves oxidative stability [10,11]. Dry-cured ham products are another type of meat products with long shelf-life that have been produced by various cultures worldwide for a long time. Products such as Presunto (Portugal), Prosciutto (Italy), and Jamón (Spain) are some examples of dry-cured ham products in Europe. The processing steps used for dry-cured ham include raw meat selection, salting, post-salting rest, dry-curing and aging or ripening; the last step is optional and applied to higher quality and more luxurious products. A key factor here is the high additive amounts (salt + nitrifying agents) used, which are usually between 35 and 90 g/kg of raw meat [12]. There are also heat-treated modern meat products which are produced mostly industrially such as Fiambre (Portugal) or Bierschinken (Germany). These products are produced with similar methods, mainly in industrial settings. Generally, the first step is mincing meat and fat at the appropriate ratios, then mixing them with additives, salt and spices. Afterwards, the batter is stuffed in casings (usually artificial), which are then heated for medium to long periods of time.

Red meat, particularly processed products of red meat, have been almost consistently attributed to many diseases, such as several types of cancer, cardiovascular diseases and metabolic problems [13,14]. However, there are studies suggesting otherwise [15,16], and stating that the cooking methods and the amount consumed are more important factors in play. In short, seemingly processed red meat products carry higher health risk factors compared to unprocessed fresh meat. A very effective method to fully and comprehensively evaluate these products and their health effects, and make comparisons, is Risk–Benefit Assessment.

Risk–Benefit Assessment (RBA) is an evaluation methodology that aims to assess both negative and positive health effects of a food constituent, product (whole food) or diet, by integrating nutritional, toxicological and microbiological aspects to reach a holistic view on a nutrition-related matter [17]. One of the most important advantages of RBA is the combined view of beneficial and adverse health effects with a more realistic and complete perspective, which is usable for policies related to public health and nutrition [18]. RBA can be performed on various levels; namely, food components and constitutes, whole food products, complete food groups or whole diets. This methodology evaluates combined microbiological, chemical and toxicological hazards, and nutritional aspects of the subjects. RBA can also have different qualitative, semi-quantitative and quantitative approaches [19], and can be a great tool to assess many ambiguous situations related to food, such as myths or common mistakes related to health effects of certain food-related subjects.

There are several studies using RBA methodology to assess various foods, food components and diets. RBA studies regarding meat and meat products have also been performed before, which has resulted in a much better understanding of the ahealth effects of various products of this type. For instance, there are some studies investigating the benefits and risks regarding the substitution of red meat with different alternatives such as fish, insect-based or plant-based proteins, or other alternative protein sources [20,21].

In this study, the health RBA methodology was used to compare unprocessed meat as a reference scenario, with various types of processed meat products, i.e., fermented, heat-treated and dry-cured meat products as alternative scenarios, in a semi-quantitative manner, to assess the potential health impact of the consumption of these different food products.

## 2. Materials and Methods

### 2.1. Study Design

**Figure 1:**
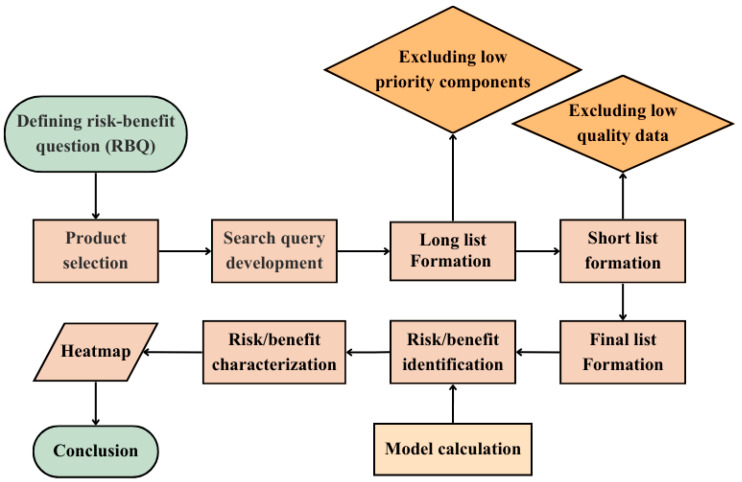
Flowchart summarizing the main steps of the study.

In this study, beneficial and adverse health effects of certain meat products have been investigated according to the RBA principles. The main steps taken in this study are summarized and shown in Figure 1.

### 2.2. The Risk–Benefit Question

This study focused on the health-related risks and benefits of the consumption of unprocessed meat and certain meat products produced with different methods. The Risk–Benefit Question (RBQ) is the research question in RBA studies which determines the scope of the assessment, the target population and the subjects to be investigated [18]. In this work, the RBQ was defined as “what would be the health impacts of consuming processed pork meat products compared to unprocessed pork meat in the Portuguese adult population?” For this purpose, according to the stepwise approach to RBA [17], different scenarios were defined (Section 2.3) and target products were selected (Section 2.4). In this study, RBA was performed on the food product level and through a semi-quantitative approach.

### 2.3. The Risk–Benefit Assessment Design

Three different alternative scenarios were designed for this study, with a reference scenario to which they were compared. Scenarios were designed as follows. (i) Reference scenario: consumption of grilled pork meat; (ii) alternative scenario 1: consumption of fermented pork meat products; (iii) alternative scenario 2: consumption of dry-cured pork meat products; and (iv) alternative scenario 3: consumption of heat-treated and additive-containing pork meat product. A certain number of products were selected for each scenario, and then the microbiological, toxicological, and nutritional aspects of the products were investigated using available data in the literature and official sources for food properties and composition databases.

For the assessment of risks and benefits of various compounds, a 4-step approach was taken. First, according to the available data, hazard and benefit identifications were performed, then hazards/benefits were characterized. Afterwards, exposure assessment was conducted according to the intake of the food products, and then calculation of compound intake was performed. In the end, the risk and benefit characterization was conducted to test the presence or absence and level of risk or benefit [22].

### 2.4. Selection of Meat Products

For the different scenarios defined in this study, the following products were selected: Chouriço and Salame (fermented meat products), Presunto (a dry-cured meat product), and Fiambre (an emulsified, heat-treated and additive-containing meat product). These products were chosen to represent the included scenarios due to their high consumption patterns among the Portuguese population within each category. The reference scenario was defined as grilled pork meat. Also, for Presunto, other closely similar products, such as the Spanish Jamón and Italian Parma ham (Prosciutto di Parma) were included.

Chouriço is a seasoned (particularly with paprika), cured or semi-cured minced pork meat product, which is subjected to fermentation and aging. Salame, another fermented minced pork meat product, is similar to Chouriço but without paprika as the main spice and with extended periods of drying leading to the firm texture. Presunto is a traditional dry-cured ham produced from whole-muscle pork (particularly Iberian pig) legs through salting and aging processes. Lastly, Fiambre is a cooked ham product produced with spices, additives and heat treatment processes.

### 2.5. Literature Reviewing, Data Collection Methods and Databases

For the present study, data were obtained from multiple sources, including published reports, legislation, and the scientific literature. When values differed across sources, we considered the minimum and maximum reported levels to capture extreme scenarios and reflect underlying variability.

#### 2.5.1. Determining the Food Components for Each Food Product

To identify the food components of interest, for each of the considered food products, a literature search was performed, following a stepwise selection approach [21]. In this method, three lists were created for each of the nutritional, microbiological and toxicological aspects, using the available data in the literature. The considered lists were the “long list”, “short list”, and “final list”. First, an extensive search on the components of each aspect was performed, contributing to the “long list”. Then, according to the level of occurrence and severity of each component, they were shortlisted (short list formation). After that, the final list was formed according to the availability and quality of the data found.

#### 2.5.2. Nutritional Data

In this study, nutritional data of the different products were extracted from the Portuguese National Food Composition Database (PortFIR) [23]. For each product that had various selectable options available in the PortFIR database the most relevant options were selected (Section S1), and final product values used in the calculations were the average of the values for each product. While for Presunto and Salame, there was only one option available in the database, there were various options available for grilled pork, Fiambre, and Chouriço.

#### 2.5.3. Microbiological and Toxicological Data

To find the appropriate data for the microbiological and toxicological aspects of the products, search queries were developed for each food product in regard to the respective aspect in question (Section S2). These were then used to retrieve data from the “Scopus” and “Web of Science” databases.

#### 2.5.4. Reference Values and Sources

The serving sizes of the investigated products were taken from the “The National Food, Nutrition and Physical Activity Survey” (IAN-AF) [24,25]. The total values, which represent the average serving size for both sexes of men and women, were considered. These serving size values are based on national and representative consumption data retrieved from the IAN-AF.

The Dietary Reference Values (DRVs) used in this study were taken from the platform “DRV Finder”, which belongs to the European Food Safety Authority (EFSA) [26]. Nutritional values for both sexes of men and women with no special conditions (e.g., pregnancy, lactating, etc.) above the age of 18 (adults) were considered for the calculations. In case there were different values suggested by the platform due to other dietary conditions, a case-specific approach was taken, which is explained in the respective results section.

The reference point values for hazard characterization (BMDL10) were taken from the “OpenFoodTox” platform, run and updated by EFSA [27]. The latest available values at the time of writing were used in the calculations.

### 2.6. RBA Model and Calculation Methods

Nutrient intake values were calculated using the food composition data from the PortFIR platform and serving size values. They were calculated using the formula below:

$$\frac{Component\;content\;\%}{100}\;\times \;Serving\;size$$

The contribution of each food product is a value stating the percentage of the contribution that a specific food product can make to the diet according to the DRVs of a specific nutritional component. This value was calculated using the formula below:

$$\frac{Average\;content\;of\;the\;component\;in\;one\;serving}{Average\;dietary\;need}\;\times \;100$$

The DRVs of lipid and linoleic acid are stated as a percentage of daily calorie intake in the EFSA database. In this study, two sets of values were calculated for the male and female sexes according to the recommended calorie intake. In these calculations, the calorie content of lipids was considered as the standard value of 9 cal/g.

For toxicological hazards having established Tolerable Daily Intake (TDI) values, a comparison between this value and the average amount of the hazardous component found in the product was performed.

For some hazardous compounds, particularly those presenting carcinogenic or genotoxic effects, and having reference points established, the Margin of Exposure (MoE) values were calculated. This was done according to the formula below:

$$\frac{Dose\;(BMDL10\;value)}{Exposure}$$

The average human body weight in this study was assumed as 70 kg.

In this study, if no research was found reporting a specific hazard or detected values, the term ‘Not Reported’, abbreviated as ‘NR’, was used. This means that, during the literature review, no studies reporting the isolation of the specific compound were retrieved. This term should not be interpreted as indicating the absence of the specific compound or resulting risks, nor as suggesting that the compound or risk cannot exist.

### 2.7. Heatmap Development and Interpretation

The heatmap was developed to visualize, in a harmonized manner, (i) nutrient contributions and (ii) hazard indicators across the evaluated food scenarios. Nutrient contributions were extracted from the data sources and expressed either as absolute amounts per serving (g) or as percentages of dietary reference values (DRVs), presented separately for males (M) and females (F), when applicable. For most nutrients, a green color scale was applied, where darker green indicates a higher contribution to the corresponding DRV or a greater absolute amount per serving. Two exceptions were implemented based on nutritional guidance: sodium and fatty acids. Sodium was coded using a yellow gradient to reflect that higher intakes are undesirable; darker yellow indicates a higher contribution to sodium intake [28]. For trans fatty acids and saturated fatty acids, color assignment was based on the presence/absence, given that the recommended intake is “As low as possible” (EFSA) [26]. Accordingly, the threshold was set to zero, and any non-zero value was coded in red (with darker red indicating higher amounts), whereas a value of zero was coded in green without a gradient. Additionally, when a nutrient contribution exceeded 100% of the DRV for a given scenario, the corresponding cell was coded in red to flag exceedance.

Hazards were visualized using two approaches reflecting the type of benchmark available: (i) hazards evaluated against a regulatory maximum level (“Reg”) and (ii) hazards evaluated using a Margin of Exposure (MoE) approach (“MoE”). For regulated hazards, cells were coded in red when values exceeded the applicable maximum level (with darker red indicating a greater exceedance), and in green when values were below the limit (with darker green indicating lower levels relative to the threshold). For MoE-based hazards, color coding followed the same red/green logic but used an MoE value of 10,000 as the decision threshold, in line with EFSA’s interpretation of MoE for substances that are genotoxic and carcinogenic [29]. MoE values ≥10,000 were coded in green, whereas values <10,000 were coded in red; color intensity was proportional to the distance from the threshold.

Where no eligible study reported quantitative values or detection for a given hazard–food combination, the cell was labeled “NR” (not reported). These cells were coded in green without gradients to distinguish absence of retrievable evidence from quantified exceedances. However, “NR” was treated as a data-availability indicator rather than evidence of absence, and was interpreted cautiously in subsequent analyses.

### 2.8. Statistical Analysis

All the statistical analyses and calculations and the creation of the heatmap in this work have been performed using Microsoft Office Excel software (Microsoft Corp., Redmond, Washington, DC, USA—Version 2510). The illustrations were constructed using the Canva online design tool (Sydney, Australia) and BioRender (November 2025, Toronto, ON, Canada) online services.

## 3. Results and Discussions

### 3.1. Selection of Hazardous Components

Through an extensive search, various microbiological and toxicological hazards were identified for each product, and a “long list” was created. After considering the occurrence and severity of each risk component, and then checking the availability and quality of the reported data in the literature, the final list of hazard components was formed. In this list, the most common, most health-threatening mutual components in the products are listed. The final list is shown in Table 1.

Meat products may contain a range of microbiological and toxicological hazards, each posing distinct health risks depending on their origin, concentration, and product processing conditions. *Listeria monocytogenes* is a foodborne bacterium frequently found in ready-to-eat meat products such as ready-to-eat, vacuum-packed cured or sliced meats. It causes listeriosis, a severe illness particularly dangerous to pregnant women, the elderly, and immunocompromised individuals, with a notably high mortality rate despite being relatively rare [30]. Histamine, a biogenic amine formed through bacterial decarboxylation of histidine during spoilage, can accumulate in semi-preserved or fermented meat products when temperature control fails [31]. Though more common in fish, its presence in meat is possible, which can lead to scombroid poisoning, with symptoms like flushing and headaches [32]. Nitrites, widely used as curing agents in processed meats such as sausages and dry-cured ham, help prevent microbial growth and improve color but can form carcinogenic nitrosamines upon reacting with amines during digestion or cooking [33]. Nitrosamines were not assessed directly, since in the European Union, the maximum permitted levels set for nitrites in processed meat products already reflect risk-management measures aimed at minimizing nitrosamine formation while ensuring product safety.

Mycotoxins, such as aflatoxins and ochratoxin A, can enter the meat chain indirectly through contaminated animal feed, or form in the product due to the growth of producing fungi on the surface or other parts of the meat product during process stages such as ripening. Aflatoxins may accumulate in organ meats and are well-established hepatocarcinogens, contributing to liver cancer risk, especially with long-term exposure [34]. Aflatoxins are recognized by the International Agency for Research on Cancer (IARC) as a Group 1 carcinogen (compounds with sufficient evidence showing they are carcinogenic to humans) due to its strong carcinogenic potential [35]. Polycyclic aromatic hydrocarbons (PAHs), particularly the PAH4 subset, are formed during incomplete combustion and are often present in smoked or grilled meat products. These compounds are genotoxic and have been associated with gastrointestinal cancers due to their ability to bioaccumulate in fat tissues [36]. Among these, benzo[a]pyrene serves as a key marker for PAH contamination. It is commonly found in charred (burned) or heavily smoked meats and is recognized by IARC as a Group 1 carcinogen [37]. While modern meat safety regulations reduce the frequency of severe exposure, these hazards remain significant public health concerns due to their persistence in global meat production and processing practices.

### 3.2. Risk Identification and Characterization

The identified hazardous risk components are listed and characterized in Table 2. *L. monocytogenes*, histamine and nitrite are evaluated according to their regulated amount, and for aflatoxins, ochratoxin A, PAH4, and benzo(a)pyrene, their MoE was calculated, using the intake amount and available reference points.

No *L. monocytogenes* presence was reported in the retrieved sources for grilled pork and Chouriço, for the reference and one of the fermented meat products considered, respectively. For the grilled pork, this is not a surprising result, since this product is usually bought fresh and prepared freshly at high temperatures. However, while Chouriço is a product that has a long duration of ripening and fermentation and needs a lot of manual handling during production, the absence of this pathogen is an interesting detail. It might be due to the fermentation process applied to Chouriço, which has been shown to have protective effects against other microorganisms, including pathogens [53]. More research is necessary to determine the actual risk of *L. monocytogenes* in Chouriço, as these results do not prove the absence of risk. Oppositely, while Salame is also a fermented product, *L. monocytogenes* was reported in this product, in high concentrations. This amount of *L. monocytogenes* has been isolated from a Salame product produced in Ecuador, and it might have been due to a different production regulation and/or regulatory enforcement, which could result in a different quality, although the product is similar to the Portuguese version. Another reason for this finding might be due to deviations from the regulations at the point of sale or during storage [48]. Another study also demonstrated the presence of *L. monocytogenes* in Salame and in Fiambre products produced in Brazil with different concentrations. The only regulation regarding *L. monocytogenes* encountered in Brazil is that the bacterium should not be present in 25 g of ready-to-eat products [42], which is similar to the established standard regulation in the European Union [54]. No study isolating this pathogen in these products in any country in Europe was encountered.

The histamine content of grilled pork and Chouriço are similar. This is surprising since histamine is usually more abundant in products that are fermented (due to bacterial activity), aged and stored for long periods of time [31]. Grilled pork does not have any of these conditions, but there are studies suggesting that grilling can increase the amount of histamine in food, which might be due to the loss of moisture increasing the concentration of the compound [32,38]. Similar to Chouriço, since there are fermentation and/or aging processes applied to Salame and Presunto, the content of histamine is also high. However, while both Salame and Chouriço are fermented products, there is a significant difference between the histamine contents of the products, with Salame containing elevated levels. This can be a result of many factors, including differences in formulations and spices (e.g., the inhibitory effect of antimicrobial compounds in spices), microbial load and starter cultures (e.g., the presence of biogenic amine-producing bacteria), fermentation conditions (e.g., humidity, temperature), production conditions (e.g., risk of contamination, smoking), and storage conditions (e.g., temperature, humidity, etc.) [31,55,56,57]. The use of microorganisms with higher production levels of biogenic amines in Salame, or the use of higher amounts of salts or spices with the ability to limit the bacterial activities (due to antimicrobial properties) including biogenic amine synthesis in Chouriço, can result in a different histamine content of the products. It has been shown in previous studies that microflora, fermentation conditions, and the recipe of the products can drastically affect the physicochemical properties of the fermented sausages, especially the biogenic amine content [58,59]. In conclusion, this seems to be a matter of production conditions, raw material and other factors rather than product type, meaning it would not be correct to suggest levels of histamine are always higher in Salame than Chouriço. There are studies demonstrating immense differences in the level of histamine and biogenic amines as a whole, in products produced in various places or with different raw materials but otherwise similar products [60,61]. Since there are no processes inducing histamine accumulation involved in the production of Fiambre, such as aging or fermenting, low levels of histamine in this product are expected.

Nitrite is an additive usually used in processed meat products to control microbial growth (especially *Clostridium botulinum*), and add specific sensorial attributes (color, taste, and smell) to the product [62]. Hence, no amount of nitrite being reported for grilled pork is expected. As for the processed meat products, lower levels of nitrite are reported for fermented products (Chouriço and Salame), while much higher levels were reported for the non-fermented Fiambre and Presunto. The reason for this can be the fact that fermentation and the presence of microorganisms in the food matrix can have a strong inhibiting effect on pathogenic or spoilage bacteria that otherwise must be inhibited through the addition of nitrites. There are studies investigating the potential of fermentation and starter culture engineering to develop versions of these products with significantly lower amounts of salt and nitrites or other usual preservatives [63,64,65]. It has been discussed that salt reduction, even in miniature amounts, can lower water activity and consequently significantly deteriorate microbial quality of products such as Chouriço [66]; hence, engineering the fermentation process to achieve suitable microbial quality is highly important. Also, fermenting microorganisms present in the food can decrease the initial levels of added nitrites during fermentation [62,67]. This would create the option of having meat products with lower levels of nitrite, which can be a hazardous compound and a precursor to carcinogenic compounds [68]. Plus, it can open the door for developing functional and probiotic meat products with additional health benefits [69].

The mycotoxins considered in this study (aflatoxins and ochratoxin A) are present in products that are aged according to their production methods. These compounds are produced by fungi, such as *Aspergillus flavus*, *Aspergillus westerdijkiae*, and *Penicillium nordicum*, which have contaminated the product through three different pathways—contaminated spices and other raw materials, the presence of these fungi on the surface of meat products, and the carry-over effect from farm animals exposed to contaminated feed [70,71]. Mycotoxins are capable of having adverse health effects in the human body [72]. Although no eligible reports were retrieved describing mycotoxin occurrence in the non-ripened/non-dry-cured products assessed (i.e., Fiambre and grilled pork), this should not be interpreted as evidence of absence. Nevertheless, given their typical processing and storage conditions, these products are unlikely to provide the environmental prerequisites for fungal growth and de novo mycotoxin production, suggesting that any potential occurrence would more plausibly reflect carry-over from contaminated ingredients rather than formation during processing. On the other hand, these compounds have been reported to be isolated from Presunto and Salame, with Salame containing the highest amount. This is thought to largely depend on the manufacturing practices and environmental condition of production, which would cause high amounts of mycotoxin production in Salame [51]. However, this does not explain why there have been no reports of mycotoxin being isolated from Chouriço, since the production conditions of both products are similar, which might be due to lack of research on this matter for this product.

PAH4 in general, and particularly benzo(a)pyrene, are compounds usually found in food products with smoking or direct wood fire cooking processes in their manufacturing methods [73]. These compounds have been shown to have various adverse health effects [36]. The absence of reports detecting these compounds in Fiambre and Presunto likely reflects that these products are typically not produced using smoking processes, which are a primary source of PAH formation. Nonetheless, this lack of retrieved evidence should not be interpreted as proof of absence; PAH4 may still occur (e.g., through raw-material contamination or processing variability), and further targeted studies are needed to characterize its occurrence in these products. As for other products, it is possible to state that if they are smoked or cooked over direct wood fire, such as grilled pork, or the occasional smoking of Salame, they would probably contain these compounds. It is important to note that this is, again, highly dependent on production practices. For instance, meat products produced in two different regions of Portugal (Alentejo and Trás-os-Montes) demonstrated highly varied amounts of PAHs while smoking was used in both regions, reflecting the diverse practices in the production of very similar or the same products [74].

It is important to note that data regarding the risk factor content of these products, particularly Presunto, Salame and Chouriço, which are traditional products, demonstrate high variability according to the production region, traditional methods of production, various practices and steps during processing, and many other factors. Hence, while the values listed in Table 2 are values present in the literature and are realistic numbers concluded from real-world products, generalizing these findings as the typical content of risk factors in all of these products around the world would introduce some uncertainty.

Also, the data available in the literature that were used for calculations in this semi-quantitative study have limitations. For instance, there are gaps in the data regarding the toxicological values of some products according to specificities of the processing practices, the type of raw materials, and the type of fermentation (starter culture or spontaneous). Another gap is regarding the microbial content of some products. For instance, the absence of *L. monocytogenes* in Chouriço does not confer that this pathogen cannot exist in this food—it means that this has not been reported, and under unsuitable processing conditions, the food might easily be contaminated. Also, the absence of this pathogen in grilled pork is highly dependent on cooking method and temperature. In this study, the minimum and maximum values found in the literature have been included for the selected risk factors, to try and represent the extreme potential values.

### 3.3. Benefit Identification and Characterization

Formatting the final list of benefits that contained the nutrients present in these products was conducted following a complete food composition listing according to the reference values (long list), followed by selecting the most abundant mutual nutrients in these products. This created the short list, which was then simply turned into the final list, since all the data taken from PortFIR was of adequate quality and availability. The chosen nutritional components for the benefit assessment are summarized in Table 3. This table also summarizes the benefits of alternative and reference scenarios.

The protein contents of the products are mostly similar, except grilled pork, which has a higher protein content. This can be due to the fact that the other products (except Presunto) are not whole-muscle meats and are prepared from a combination of muscle meat and other raw materials, such as spices. Moreover, different meat cuts contain different protein contents, which can be a factor at play here [75]. Also, the manufacturing processes for meat products might have reducing effects on the protein content [76], while grilled pork is directly and freshly prepared.

Trans and saturated fatty acids are hazardous compounds which are suggested by EFSA to be consumed in amounts as low as possible. While all these products contain some amounts of these two lipid types, some of them, i.e., Fiambre and Presunto, contain comparably lower amounts. This is probably due to the type of meat selected for the production of these products and their respective manufacturing methods. For instance, as it can be seen from the lower lipid content of Presunto, the type of meat selected for this product is usually muscle pieces with lower contents of fatty tissue, which would result in lower lipid-related compounds such as trans and saturated fatty acids [77]. Also, it has been observed in previous studies that throughout the production of dry-aged sausages, particularly smoked products, such as Chouriço and Salame, the ratio of unsaturated to saturated fatty acids (P/S) decreases [78], which lowers the health concerns regarding these products.

In terms of minerals, although all products have some levels of contribution to the daily necessary intake of minerals, there are plant-based sources that are superior in many mineral contents such as calcium and magnesium [79]. The only exception would be iron and zinc, which demonstrates a relatively high content in Presunto, Chouriço and Salame. This can be due to the nature of these products. Red meat has higher amounts of iron compared to many main food groups [80]. This makes these products suitable sources of nutritional iron. However, due to other risks present in these products, a diversification of iron sources could be of equal importance.

### 3.4. Heatmap-Based Risk–Benefit Assessment

To combine the risk and benefit assessments demonstrated in the last sections and to integrate both perspectives, a heatmap was created including all the alternative scenarios and the reference scenario (Table 4). In this heatmap there are two main categories, nutrients (benefits) and hazards (risks). The version of this heatmap with all the value numbers included can be found in Table S1.

The heatmap demonstrates that the protein content of none of the products exceeds the suggested protein consumption limit determined by EFSA (0.66 g/kg body weight per day), considering the reference serving sizes for each product [26]. While all products contribute substantially to the daily protein intake, the highest level of protein belongs to grilled pork. This suggests these products are proper and suitable sources of protein for consumers. Also, although all these products are suitable protein sources, grilled pork seems to have lower levels of risk with higher levels of protein content, making it the better option for protein absorption.

As for lipid contents, the products demonstrated substantial levels which also did not exceed the limits set by EFSA (20% of calorie intake) [26]. Along with the higher lipid content in these red meat products, naturally, saturated and trans fatty acids also exist. Grilled pork has demonstrated the highest content of both groups. And the lowest levels can be seen in Fiambre and Presunto, which is thought to be due to the lower amount of total lipid content of the selected meat pieces and the production process. However, trans and saturated fatty acids are suggested to be consumed in amounts as little as possible by EFSA guidelines. Hence, almost all products cannot be considered suitable, except Presunto and Fiambre in terms of trans fatty acid content. The linoleic acid contents of the products are in a suitable range, and none exceeds the EFSA consumption limits (4% of daily calorie intake). Linoleic acid has been demonstrated to have various positive health effects [81]. However, due to the low contribution of each food to dietary linoleic acid, considering these food products as good sources of linoleic acid would be an incorrect conclusion.

Among minerals, sodium presented the highest amounts in all processed traditional products, i.e., Chouriço, Presunto and Salame, and the lowest amount in the grilled pork and Fiambre. Although these values do not exceed the daily consumption limits defined by EFSA (2000 mg/day [26]), only one serving of these traditional products contributes to approximately 25% of the daily consumption amount, which is a substantial contribution to the daily intake of sodium. This would present high levels of risk concerning other sources of sodium in the daily diet, particularly for high consumers and the consumption of multiple servings per day (please see Table S1 for a numbered heatmap). High levels of sodium consumption have been attributed to various health problems including cardiovascular diseases [82]. Hence, consumption of products that contain high levels of sodium must be done cautiously. High sodium content is also one of the main reasons for the attribution of processed meat products with many health problems [83]. There have been studies researching the potential of fermentation to reduce the salt needed in meat products [63]. This is an immense potential that should be investigated in more detail and applied in industry. Regarding other essential minerals listed in the heatmap, grilled pork contains the highest amount for almost every mineral, with Chouriço and Salame following. No product exceeds the tolerable upper intake level. The iron content values in Chouriço and Salame are higher than those of grilled pork, which can be attributed to the fermentation process that could potentially increase the iron content and its bioavailability [84]. Also, the process of these products reduced the water content compared to fresh meat (grilled pork), which can also explain the higher levels of iron in these products. Overall, red meat and its products are suitable sources of dietary iron, and here, except Fiambre, all scenarios contribute meaningfully to the dietary iron absorption. As for other minerals, considering the low levels of contribution of these foods to the DRV of the respective minerals, considering them as adequate sources for these essential minerals would not be correct.

There have been a few cases where *L. monocytogenes* has been found to be present in Fiambre, Presunto, and Salame [45,48]. However, for Presunto, the concentration of the microorganism has been lower than the maximum limit (100 CFU/g) [54], and for the other two products, this value was found to be above the limit (see the numbered heatmap in Table S1). For histamine, although reported to be present in all products, the levels of the compound have been found to be lower than the regulated limit (150 mg/kg) [29]. Only Salame has been reported to contain amounts of histamine higher than the limit. As for the nitrite content of the products, there is only grilled pork with no reports stating the isolation of nitrites from it, which is expected since this compound is used in processed meat products to ensure safety and improved color. Nitrite, however, while being effective against dangerous pathogens in meat products and having other functionalities, also has been demonstrated to have adverse health effects on the human body and to cause problems such as cancer [33]. Hence, the levels of this food additive must be under control and checked. As can be seen in the heatmap, the levels of nitrite in Presunto and Fiambre are the highest among the products. The amount of nitrite used in these products is the maximum limit allowed for this additive to exist in meat products [85]. However, this is not the case in Salame and Chouriço, which can be attributed to the fermented nature of these products, which might have decreased the need for nitrites to ensure safety. There have been many studies investigating the potential of fermentation (selective fermentation, culture development, etc.) to decrease the need for additives such as salts, nitrites, etc., in fermented meat products such as Chouriço and Salame [62,63,64,65]. This is highly possible due to the immense potential of microorganisms to alter their environment, which can be utilized for this purpose. Ochratoxin A, a mycotoxin, has been reported in Salame and Presunto with higher levels in Salame. Since these two products are ripened during production, unsuitable storage conditions including temperature, humidity, etc., can facilitate the growth of unwanted fungi, especially on the surface of the food. PAH4 and benzo(a)pyrene are compounds usually found in foods that have been smoked or cooked over direct fire, especially wood fire. These compounds are considered carcinogenic. PAH4 has been reported in grilled pork, Chouriço, and Salame. Grilled pork contains these compounds due to the cooking method applied. Salame and Chouriço contain these if they are smoked as a part of their production process.

It should be emphasized that the regional and technological variability in curing and fermentation (e.g., curing salt concentrations, fermentation/ripening temperature and humidity, and smoking intensity/duration) can substantially shift the levels of the considered food components such as nitrite, biogenic amines, PAHs and mycotoxins. Therefore, the obtained results should be read as representative of the production conditions reflected in the underlying data sources, and the heatmap as a comparative tool under defined assumptions, rather than a universally applicable ranking of product risk.

### 3.5. Study Strengths and Limitations

To the best of our knowledge, this study is the first to use a heatmap-based semi-quantitative RBA methodology to investigate the health benefits and risks of meat products and compare various processing methods. Particularly, through the validated and objective quantitative tools available in this methodology, the resulting data allows for more decisive and clear conclusions, suitable and useful for both health and food policy-making bodies and regular consumers. The comparative design of the RBQ and the study itself with different scenarios facilitates more comprehensive conclusions. The comprehensive approach employed in the study and the search strings used for data retrieval improve the reproducibility of the study, strengthening the functionality for various use cases. Additionally, the overall research question of the study is highly relevant considering the present public health issues worldwide. Despite these notable strengths, several limitations should be acknowledged, which were confronted in different aspects of the research.

The first limitation of this study is the data availability in the literature. The number of studies investigating these subjects is limited in terms of diversity, data quality and availability, and molecular mechanisms involved. For instance, there were studies that mentioned the isolation of certain compounds, especially hazardous components, from products, but reported no values. In future research, improvement in the reporting of results would facilitate the precision of future RBAs.

Fermentation also forms new bioactive compounds in food, many of which have been suggested to have beneficial health effects [86,87]. Fermentation also has the potential of increasing the bioavailability and bioactivity of various nutritional compounds in the fermented food [88]. However, due to the lack of evidence and research on the effects of fermentation on these products, it was not possible to also include these components in the benefits assessment. Various aspects of fermented foods, particularly aspects concerning health-promoting bioactive compounds, should be addressed in future research. This would facilitate more in-depth and detailed RBA investigations. Also, due to the power of fermentation and starter culture optimization and engineering, the development of high-protein healthy meat-based functional foods would be possible. Currently, this potential of fermentation is being realized in plant-based foods [89,90]. As stated before, fermentation also has the potential to facilitate the development of novel and healthier meat products with reduced levels of preservatives and additives.

The alternative scenario products investigated in this study are mostly traditionally manufactured products with many variations in the production processes, which might subject the components’ profile to change. For instance, the level of smoking can alter the PAH content of the product; the starter culture, or duration of fermentation process, can drastically modify the end-product, and its quality and composition. Hence, there should be deeper research into the different variants of each product to reach a more generally applicable conclusion.

## 4. Conclusions

RBA studies investigating meat and meat products could deepen our understanding of the health effects associated with these foods, which can be diverse in terms of the health concerns they pose. This would also strongly facilitate regulatory bodies’ activities to give more informed suggestions to the public and help people in their everyday diet decisions.

In terms of results, this semi-quantitative RBA study provides a comprehensive assessment of meat products from nutritional and toxicological standpoints. This study demonstrates that meat and meat products are adequate sources of many macronutrients, such as lipids and proteins, and micronutrients, such as iron and zinc. However, health concerns related to meat, especially processed meat products, are also rightful concerns. Among the selected products, grilled pork presented a favorable nutritional value with a relatively lower content of hazardous compounds when compared to processed meat products. The only exception is higher levels of trans and saturated fats in grilled pork compared to other products. In contrast, processed meat products with high concentrations of salt, nitrogen-based preservatives and additives, trans and saturated fatty acids, and many chemical hazards that are process contaminants, such as histamine and PAHs, might have the potential of causing health problems. Salame demonstrated relatively high risks regarding the presence of *L. monocytogenes*, histamine and ochratoxin A. Chouriço, the other fermented product investigated in the study, while carrying fewer risk factors compared to Salame, still presented some levels of risk due to high concentrations of salt and containing some amounts of nitrite. Fiambre demonstrated fewer microbiological and toxicological risks but still contained high levels of nitrite due to the maximum allowed content in regulations. Presunto also similarly demonstrated high levels of salt and nitrite, while also presenting the risk of carrying mycotoxins, which can be attributed to the ripening stage of the production process.

In short, according to these findings, it is possible to conclude that, in a scenario-specific manner, freshly prepared meat products can be healthier options, as long as the cooking process does not introduce harmful contaminants. After that, products fabricated with modern methods, such as Fiambre, seem to be the safest meat products compared to traditional products, due to high levels of preservatives, process contaminants, and production standards. Traditional products, on the other hand, can present immense potential for even healthier and novel products if the processes are investigated scientifically and production takes place with modern methods and equipment. Also, the highest risks with these products currently are high levels of preservatives, sodium containing salts, smoking applications, insufficient production control, improper fermentation conditions, wrong storage conditions, and problematic raw materials. These are some of the most important areas policy-making processes can concentrate on.

These findings indicate the high relevance of tools supporting consumers making healthier choices. Tools like Risk–Benefit Assessment can be adapted and transformed for facilitating the understanding of consumers to assist them in modifying their everyday diets for healthier nourishment. Health concerns regarding these products can also be addressed, and the products can be improved by utilizing techniques such as fermentation with modern methods, e.g., tailoring and engineering strains and fermentation conditions; or by investigating novel, alternative and multi-purpose salts and/or spices with various functionalities such as antimicrobial properties. These future directions will address the issues found and mentioned in this study and facilitate solving these problems. For instance, future research directions can be useful to decrease the sodium and/or nitrate content of the products, or engineered fermenting strains can facilitate the process to reduce the risk of mycotoxins and/or pathogens. Additionally, future RBA models should be developed to include bioactives present in the products, especially metabolites resulting from fermentation, and to consider regional variations in the production of these products to increase the generalizability of the findings and potentially find better and healthier production processes. Also, RBA models with weight distribution models, including multiple serving data, real-world surveys, and high consumption levels according to the importance of the components, can be very useful for more generalized and decisive conclusions.

## Figures and Tables

**Table 1:** Final list of hazardous components.

Hazard Type	Hazardous Component
Microbiological	*L. monocytogenes*
Toxicological	Histamine
	Nitrite
	Aflatoxins
	Ochratoxin A
	Polycyclic Aromatic Hydrocarbons 4 (PAH4)
	Benzo(a)pyrene

**Table 2:** Risk factors in comparison with regulation or margin of exposure (MoE).

Food	Component	Component Value	Regulated Amount/Reference Point	Intake (mg/kg bw ^1^)	Margin of Exposure	Reference
Grilled Pork	*L. monocytogenes*	NR ^2^	100 CFU ^3^/g	0	NA ^2^	-
Grilled Pork	Histamine	4.10 mg/kg	150 mg/kg	0.00305	NA	[38]
Grilled Pork	Nitrite	NR	0.07 mg/kg	0	NA	-
Grilled Pork	Aflatoxins	NR	0.0004 mg/kg bw/day	0	NR	-
Grilled Pork	Ochratoxin A	NR	0.01 mg/kg bw/day	0	NR	-
Grilled Pork	PAH4	0.11 mg/kg	0.34 mg/kg bw/day	0.00008	4011.68	[39]
Grilled Pork	Benzo(a)pyrene	0.03 mg/kg	0.07 mg/kg bw/day	0.00002	2980.57	[39]
Chouriço	*L. monocytogenes*	NR	100 CFU/g	0	NA	-
Chouriço	Histamine	5 mg/kg	150 mg/kg	0.00150	NA	[38]
Chouriço	Nitrite	25 mg/kg	0.07 mg/kg	0.00750	NA	[40]
Chouriço	Aflatoxins	NR	0.0004 mg/kg bw/day	0	NR	-
Chouriço	Ochratoxin A	NR	0.01 mg/kg bw/day	0	NR	-
Chouriço	PAH4	0.70 mg/kg	0.34 mg/kg bw/day	0.00021	1613.29	[39]
Chouriço	Benzo(a)pyrene	0.00180 mg/kg	0.07 mg/kg bw/day	0.000001	129,990.71	[41]
Fiambre	*L. monocytogenes*	800 CFU/g	100 CFU/g	0	NA	[42]
Fiambre	Histamine	0.27 mg/kg	150 mg/kg	0.00008	NA	[43]
Fiambre	Nitrite	100 mg/kg	0.07 mg/kg	0.03	NA	[44]
Fiambre	Aflatoxins	NR	0.0004 mg/kg bw/day	0	NR	-
Fiambre	Ochratoxin A	NR	0.01 mg/kg bw/day	0	NR	-
Fiambre	PAH4	NR	0.34 mg/kg bw/day	0	NR	-
Fiambre	Benzo(a)pyrene	NR	0.07 mg/kg bw/day	0	NR	-
Presunto	*L. monocytogenes*	22.50 CFU/g	100 CFU/g	0	NA	[45]
Presunto	Histamine	6.05 mg/kg	150 mg/kg	0.001815	NA	[46]
Presunto	Nitrite	100 mg/kg	0.07 mg/kg	0.03	NA	-
Presunto	Aflatoxins	NR	0.0004 mg/kg bw/day	0	NR	-
Presunto	Ochratoxin A	0.08 mg/kg	0.01 mg/kg bw/day	0.000024	400.88	[47]
Presunto	PAH4	NR	0.34 mg/kg bw/day	0	NR	-
Presunto	Benzo(a)pyrene	NR	0.07 mg/kg bw/day	0	NR	-
Salame	*L. monocytogenes*	3802 CFU/g	100 CFU/g	0	NA	[48]
Salame	Histamine	235.69 mg/kg	150 mg/kg	0.07071	NA	[49]
Salame	Nitrite	14.75 mg/kg	0.07 mg/kg	0.00093	NA	[50]
Salame	Aflatoxins	NR	0.0004 mg/kg bw/day	0	NR	-
Salame	Ochratoxin A	103.69 mg/kg	0.01 mg/kg bw/day	0.01556	0.62	[51]
Salame	PAH4	6.51 mg/kg	0.34 mg/kg bw/day	0.00132	258.46	[52]
Salame	Benzo(a)pyrene	0.89 mg/kg	0.07 mg/kg bw/day	0.00016	428.13	[52]

^1^ bw: Body Weight. ^2^ NR: Not Reported, and NA = Not Applicable. ^3^ CFU: Colony Forming Units. If no eligible studies reporting a given compound were retrieved, it is indicated as “Not reported” (NR). This denotes a lack of evidence in the retrieved literature and should not be interpreted as evidence of absence or as implying that the compound cannot occur.

**Table 3:** Comparison of nutritional values with suggested DRVs.

Food	Component	Value in Serving	DRVs	Contribution of the Food (%)
Grilled Pork	Protein	8.53 g	46.2 g	18.46
Grilled Pork	Lipid	21.35 g	M ^1^ 55.55 g F ^1^ 44.44 g	M 8.00 F 10.01
Grilled Pork	Total saturated fatty acids	7.67 g	As low as possible	NA ^2^
Grilled Pork	Linoleic acid	2.59 g	M 11.11 g F 8.89 g	M 0.97 F 1.22
Grilled Pork	Total trans fatty acids	0.18 g	As low as possible	NA
Grilled Pork	Sodium	101.40 mg	2000 mg	5.07
Grilled Pork	Potassium	144.82 mg	3500 mg	4.14
Grilled Pork	Calcium	17.68 mg	750 mg ^3^	2.36
Grilled Pork	Phosphorus	43.16 mg	550 mg	7.85
Grilled Pork	Magnesium	32.76 mg	M 350 mg F 300 mg	M 9.36 F 10.92
Grilled Pork	Iron	0.44 mg	M 6 mg F 7 mg ^4^	M 7.37 F 6.31
Grilled Pork	Zinc	0.94 mg	M 10.13 mg F 8.23 mg	M 9.61 F 11.78
Chouriço	Protein	4.19 g	46.2 g	9.07
Chouriço	Lipid	9.25 g	M 55.55 g F 44.44 g	M 3.47 F 4.34
Chouriço	Total saturated fatty acids	3.19 g	As low as possible	NA
Chouriço	Linoleic acid	0.91	M 11.11 g F 8.89 g	M 0.34 F 0.43
Chouriço	Total trans fatty acids	0.03	As low as possible	NA
Chouriço	Sodium	517 mg	2000 mg	25.88
Chouriço	Potassium	60.90 mg	3500 mg	1.74
Chouriço	Calcium	5.57 mg	750 mg	0.74
Chouriço	Phosphorus	50.40 mg	550 mg	9.16
Chouriço	Magnesium	4.41 mg	M 350 mg F 300 mg	M 1.26 F 1.47
Chouriço	Iron	0.55 mg	M 6 mg F 7 mg	M 9.10 F 7.80
Chouriço	Zinc	0.65 mg	M 10.13 mg F 8.23 mg	M 6.68 F 8.19
Fiambre	Protein	3.21 g	46.2 g	6.94
Fiambre	Lipid	0.97 g	M 55.55 g F 44.44 g	M 0.36 F 0.45
Fiambre	Total saturated fatty acids	0.32 g	As low as possible	NA
Fiambre	Linoleic acid	0.12 g	M 11.11 g F 8.89 g	M 0.04 F 0.05
Fiambre	Total trans fatty acids	0	As low as possible	NA
Fiambre	Sodium	196.70 mg	2000 mg	9.84
Fiambre	Potassium	69.30 mg	3500 mg	1.98
Fiambre	Calcium	2.24 mg	750 mg	0.30
Fiambre	Phosphorus	56.70 mg	550 mg	10.31
Fiambre	Magnesium	3.29 mg	M 350 mg F 300 mg	M 0.94 F 1.10
Fiambre	Iron	0.7 mg	M 6 mg F 7 mg	M 2.33 F 2.00
Fiambre	Zinc	1.4 mg	M 10.13 mg F 8.23 mg	M 2.95 F 3.61
Presunto	Protein	5.25 g	46.2 g	11.36
Presunto	Lipid	2.69 g	M 55.55 g F 44.44 g	M 1.01 F 1.26
Presunto	Total saturated fatty acids	0.86 g	As low as possible	NA
Presunto	Linoleic acid	0.25 g	M 11.11 g F 8.89 g	M 0.09 F 0.12
Presunto	Total trans fatty acids	0	As low as possible	NA
Presunto	Sodium	539.70 mg	2000 mg	26.99
Presunto	Potassium	121.80 mg	3500 mg	3.48
Presunto	Calcium	4.83 mg	750 mg	0.64
Presunto	Phosphorus	42.00 mg	550 mg	7.64
Presunto	Magnesium	8.61 mg	M 350 mg F 300 mg	M 2.46 F 2.87
Presunto	Iron	0.42 mg	M 6 mg F 7 mg	M 7.00 F 6.00
Presunto	Zinc	0.82 mg	M 10.13 mg F 8.23 mg	M 6.68 F 8.19
Salame	Protein	4.10 g	46.2 g	8.86
Salame	Lipid	7.90 g	M 55.55 g F 44.44 g	M 2.96 F 3.70
Salame	Total saturated fatty acids	2.71 g	As low as possible	NA
Salame	Linoleic acid	0.78 g	M 11.11 g F 8.89 g	M 0.29 F 0.36
Salame	Total trans fatty acids	0.02 g	As low as possible	NA
Salame	Sodium	483.00 mg	2000 mg	24.15
Salame	Potassium	29.40 mg	3500 mg	0.84
Salame	Calcium	5.25 mg	750 mg	0.70
Salame	Phosphorus	42.00 mg	550 mg	7.64
Salame	Magnesium	4.62 mg	M 350 mg F 300 mg	M 1.32 F 1.54
Salame	Iron	0.48 mg	M 6 mg F 7 mg	M 8.05 F 6.90
Salame	Zinc	0.90 mg	M 10.13 mg F 8.23 mg	M 9.27 F 11.36

^1^ M = male, F = female. ^2^ NA = Not Applicable. ^3^ The calcium need for people 25 years of age and above was considered. ^4^ The iron need for premenopausal women was considered.

**Table 4:** The heatmap summarizing the risks and benefits assessed for each scenario.

			Food Products				
Category	Components	Sex	Grilled Pork	Chouriço	Presunto	Fiambre	Salame
Nutrients (g per serving/% of food contribution per serving)	Proteins	FM					
	Lipids	M					
		F					
	Fatty acids, total saturated (g)	FM					
	Linoleic acid	M					
		F					
	Fatty acids, total trans (g)	FM					
	Sodium	FM					
	Potassium	FM					
	Calcium	FM					
	Phosphorus	FM					
	Magnesium	M					
		F					
	Iron	M					
		F					
	Zinc	M					
		F					
Hazards (Regulation/Margin of exposure)	*L. monocytogenes* (Reg)	FM					
	Histamine (Reg)	FM					
	Nitrite (Reg)	FM					
	Ochratoxin A (MoE)	FM					
	PAH4 (MoE)	FM					
	Benzo(a)pyrene (MoE)	FM					

Nutrients: green scale (darker = higher contribution); sodium uses a yellow scale (darker = higher); trans/saturated fats: 0 = green, >0 = red (darker = higher); >100% of the DRV = red. Hazards: Regulated (Reg)—red above the limit/green below (darker = farther from the limit); MoE—threshold 10,000, green ≥ 10,000 and red < 10,000 (darker = farther from the threshold).

## Data Availability

No new data were created or analyzed in this study. Data sharing is not applicable to this article.

## Supplementary Materials

The following supporting information can be downloaded at https://www.mdpi.com/article/10.3390/foods15040661/s1.

## Abbreviations

The following abbreviations are used in this manuscript:

RBA	Risk–Benefit Assessment
RBQ	Risk–Benefit Question
EFSA	European Food Safety Authority
CFU	Colony Forming Unit
DRV	Daily Reference Value
TDI	Tolerable Daily Intake
PAH4	Polycyclic Aromatic Hydrocarbons 4
Reg	Regulation values
MoE	Margin of Exposure
IARC	International Agency for Research on Cancer
NA	Not Applicable
NR	Not Reported
BMDL	Lower Confidence Limit
PortFIR	Portuguese Food Information Resource
DALY	Disability-Adjusted Life Years
