# Supplementary material for: A Heatmap-Based Risk–Benefit Assessment of Traditional and Processed Meat Products
Source: Foods. 2026 Feb 12;15(4):661. doi: 10.3390/foods15040661 (PMC12939101; doi:10.3390/foods15040661)
Supplement: Supplementary file 1 [file foods-15-00661-s001.zip › foods-4094600-supplementary.pdf]

Section S1

Relevant Options For Food Products

For *Fiambre*, nutritional values for “Fiambre de porco” (cooked pork ham), “Fiambre de pá” (ham, hand), “Fiambre da perna” (ham, leg) were considered and the average values were calculated and used.

For *Chouriço*, “*Chouriço* lean fat raw”, “*Chouriço*, lean, raw”, “*Chouriço*, pork meat, raw”, were the available options on the database. Similar to *Fiambre*, all these values were considered, and the average values were used for future calculations and RBA of *Chouriço*.

Lastly, for grilled pork, the reference scenario, values for “Pork, steak, grilled”, “Pork's backfat, lean and fat, grilled, no salt added” were considered and average values were calculated and used.

Section S2

Search Strings Used To Retrieve Data from the Published Literature

Microbiological Data

To find the appropriate data for the microbiological aspects of the products there were search queries developed for each food product. These were then used on "Scopus" and "Web of Science" databases.

For *Presunto*, the search query was developed and used as: "((dry-cured AND ham) OR jamon OR Presunto OR (parma AND ham)) AND (pathogen* OR microb*)". As stated before, to achieve better and more holistic results, closely similar products were also included in the search for *Presunto*.

For Chorizo, the developed search query was as follows: "(chorizo OR chouriço) AND (pathogen* OR microb*)".

For *Fiambre*, the following two queries were used. "(Fiambre OR ham) AND (monocyto* OR listeria) AND (load OR count OR amount OR Enumera* OR Quantif*) NOT (spik* OR dry-cured OR dry)", "(Fiambre OR ham) AND (pathogen* OR microb*))".

For Salami, the query "salami AND (pathogen* OR microb*)" was used to find appropriate data.

Lastly, for grilled pork, the following search query was developed "((pork AND grill* AND steak) OR (pork AND backfat AND grill*) OR (pork AND grill*) AND (pathogen* OR microb*))".

Toxicological Data

To find the appropriate data for the toxicological aspects of the products there were search queries developed for each food product. These were then used on "Scopus" and "Web of Science" databases.

For *Presunto*, the search query was developed and used as: "((dry-cured AND ham) OR jamon OR presunto OR (parma AND ham)) AND (toxi* OR harm* OR (chemical AND contamina*))".

For Chorizo, the developed search query was as follows: "(chorizo OR chouriço) AND (toxi* OR harm* OR (chemical AND contamina*))".

For *Fiambre*, the following query was used: "((Fiambre OR ham) AND (toxi* OR harm* OR (chemical AND contamina*))".

For Salami, the query "salami AND (toxi* OR harm* OR (chemical AND contamina*))" was used to find appropriate data.

Lastly, for grilled pork, the following search query was developed "((pork AND grill* AND steak) OR (pork AND backfat AND grill*) OR (pork AND grill*) AND (toxi* OR harm* OR (chemical AND contamina*))".

			Food product				
Category	Components	Sex	**Grilled pork**	***Chouriço***	***Presunto***	***Fiambre***	***Salame***
Nutrients (g per serving / % of food contribution per serving)	**Proteins**	FM	18.46	9.07	11.36	6.94	8.86
	**Lipids**	M	29.11	12.60	3.67	1.32	10.77
		F	36.39	15.77	4.58	1.65	13.46
	**Fatty acids, total saturated (g)**	FM	7.67	3.19	0.86	0.32	2.71
	**Linoleic acid**	M	24.33	8.56	2.36	1.08	7.28
		F	30.41	10.71	2.95	1.35	9.11
	**Fatty acids, total trans (g)**	FM	0.18	0.03	0.00	0.00	0.02
	**Sodium**	FM	5.07	25.88	26.99	9.84	24.15
	**Potassium**	FM	4.14	1.74	3.48	1.98	0.84
	**Calcium**	FM	2.36	0.65	0.56	0.26	0.61
	**Phosphorus**	FM	7.85	9.16	7.64	10.31	7.64
	**Magnesium**	M	9.36	1.26	2.46	0.94	1.32
		F	10.92	1.47	2.87	1.10	1.54
	**Iron**	M	7.37	9.10	7.00	2.33	8.05
		F	6.31	7.80	6.00	2.00	6.90
	**Zinc**	M	9.61	6.68	8.41	2.95	9.27
		F	11.78	8.19	10.30	3.61	11.36
Hazards (Regulation / Margin of exposure)	***L. monocytogenes* (Reg)**	FM	NR	NR	Present	Present	Present
	**Histamine (Reg)**	FM	4.10	5.00	NR	0.27	235.69
	**Nitrite (Reg)**	FM	NR	25.00	100.00	100.00	14.75
	**Ochratoxin A (MoE)**	FM	NR	NR	400.88	NR	0.62
	**PAH4 (MoE)**	FM	4011.68	1613.29	NR	NR	258.46
	**Benzo(a)pyrene (MoE)**	FM	2980.57	129990.71	NR	NR	428.13

Table S1. The heatmap (numbered) summarizing the risks and benefits assessed for each scenario
